# A Systematic Review of Home Modifications for Aging in Place in Older Adults

**DOI:** 10.3390/healthcare13070752

**Published:** 2025-03-27

**Authors:** Su-Min Cha

**Affiliations:** Department of Occupational Therapy, Kyungnam University, Changwon 51767, Republic of Korea; csm1206@kyungnam.ac.kr; Tel.: +82-055-249-6479

**Keywords:** aging in place, home modification, older adults

## Abstract

**Background/Objectives:** The aging population has increased the demand for home modifications to support aging in place. However, existing research primarily focuses on fall prevention and physical safety, leaving gaps in understanding long-term sustainability, social engagement, and cost-effectiveness. Additionally, the interaction between home modifications and health-related changes remains under-explored. This study systematically reviews home modifications, addressing these gaps by considering functional independence, quality of life, caregiving burden, and technological advancements. **Methods:** A systematic review was conducted following PRISMA 2020 guidelines. One researcher and an external expert performed study selection, data extraction, and quality assessment. Thematic analysis and narrative synthesis were applied to compare study results. **Results:** Among 20 studies, 13 (65%) confirmed the effectiveness of home modifications in fall prevention, functional independence, and cost savings. Seven studies (35%) highlighted housing accessibility and lifestyle factors. However, few studies examined personalized interventions, smart home technologies, and long-term adaptability. **Conclusions:** This study emphasizes the need for personalized, technology-driven, and long-term adaptable home modifications. Future research should explore smart home innovations, interdisciplinary approaches, and policy implementation feasibility to develop sustainable aging-in-place strategies. By adopting a holistic perspective, this study provides a new framework for advancing aging-in-place strategies.

## 1. Introduction

The rapid progression of global aging has heightened interest in the living arrangements and residential environments of older adults [1]. According to the United Nations, the proportion of individuals aged 65 and older worldwide is projected to reach 16% by 2050, with some regions surpassing 25% [1]. Additionally, the global population of individuals aged 80 and older is expected to triple by 2050, significantly increasing the demand for appropriate housing and social support systems [2]. Amid this demographic shift, the concept of aging in place (AIP) has gained substantial attention, emphasizing the ability of older adults to live independently and safely in their own homes rather than in institutionalized care settings [3]. AIP extends beyond remaining in one’s residence; it involves creating an environment that fosters physical and psychological stability, autonomy, and overall well-being [3]. A recent AARP study [4] found that over 87% of adults aged 65 and older would prefer to remain in their current home as they age. However, U.S. Census Bureau data indicate that only 10% of American homes are adequately designed for aging populations [5]. Additionally, in the Netherlands, more than 40,000 households occupied by individuals aged 65 and older are categorized as unsuitable for aging residents [6,7]. This evidence underscores that residential environments pose a major barrier to aging in place, a challenge prevalent across European nations, particularly for individuals aged 80 and older [8]. The ability of older adults to age in place is closely associated with emotional stability, social connectivity, and quality of life. At a policy level, AIP is regarded as a cost-effective strategy to reduce long-term care facility admissions and alleviate social costs [9]. However, age-related declines in physical function increase fall risk, limit mobility, and create environmental barriers, hindering independent living [10]. In response, home environment modifications (HEMs) have been proposed as a critical intervention strategy [11].

As physical and cognitive function declines, environmental barriers increasingly impact independence and safety [12]. The discrepancy between environmental demands and an individual’s functional capacity exacerbates disability [13,14], contributing to increased fall risk [15], emergency department visits [16], and transitions to long-term care facilities [17,18]. Falls occurring within the home are a major health concern, with environmental factors being a primary cause [19,20]. Previous systematic reviews have demonstrated that home modifications improve functional performance and reduce falls among older adults with various health conditions [21]. Wahl et al. (2009) [13] examined the impact of home modifications on disability-related outcomes and found that these interventions significantly enhance functional performance.

Gitlin (1998) [22] defined home environment modifications as a comprehensive strategy, including structural renovations, assistive devices, visual cues, memory aids, furniture rearrangement, removal of hazardous items, and task simplification. Occupational therapy guidelines define home modifications as interventions that adapt the environment to enhance usability, safety, and independence [23]. The home modification process involves assessment, solution implementation, training, and evaluation.

The interaction between an individual’s physical and cognitive abilities and the built environment significantly influences functional independence and quality of life. According to the environmental press theory, balance is maintained when the environment supports an individual’s capabilities. However, if environmental demands exceed functional capacity, environmental pressure increases, leading to a decline in quality of life. The physical and cognitive capabilities of older adults interact with their living environment, affecting residential satisfaction and independent living. When housing environments impose excessive demands beyond an individual’s diminished functional capacity, disability and dependence increase, necessitating tailored home modifications [24]. Home modifications are also closely linked to the person–environment–occupation (PEO) model. This model emphasizes the dynamic interaction among personal (physical, psychological, and cognitive), environmental (physical and social), and occupational (daily activity) factors, which together determine functional independence and quality of life in older adults [25]. Natalia et al. [25] particularly highlight that smart home technologies, ramp installations, and bathroom modifications are essential home adaptations that enhance independent living for older adults. These findings suggest that home modifications are a critical strategy for improving functional performance, reducing nursing home admissions, and enhancing overall quality of life.

Existing studies on home modifications for older adults have primarily focused on fall prevention and the maintenance of physical function, often providing only a partial assessment of their overall effectiveness. However, in a super-aged society, a more comprehensive approach is required—one that extends beyond physical safety to encompass functional independence, quality of life, caregiving burden, cost-effectiveness, and social participation. Unlike previous studies that examined home modifications in isolation, this study adopts a holistic perspective by integrating socioeconomic factors and technological advancements to develop practical and sustainable intervention strategies. To achieve this objective, this study employs a systematic review methodology to examine the intervention methods, effectiveness, accessibility, and policy support associated with home modifications for aging in place. Additionally, it evaluates both the short-term and long-term impacts of these modifications, providing evidence-based recommendations for customized intervention models and policy frameworks that ensure sustainability and practical applicability in an aging society.

The key research questions of this study are as follows:

(1) Primary Research Question
  - What is the comprehensive impact of home modifications on aging in place among older adults?

(2) Secondary Research Questions
  - What are the primary types and specific applications of home modifications for older adults?
  - What are the multidimensional effects of home modifications on aging in place?
  - What are the interactions and comprehensive impacts of health changes and home modifications among older adults?

This study aims to provide scientific evidence to guide policy development, healthcare interventions, and future research, ultimately contributing to the enhancement of aging-in-place strategies.

## 2. Materials and Methods

### 2.1. Study Design

Based on the PRISMA 2020 checklist, a systematic literature review was conducted on home modifications for older adults living in the community. This review identified and categorized relevant studies and synthesized their findings and differences.

The review protocol and full methodological details were preregistered in the Open Science Framework (OSF) to ensure transparency and reproducibility. The registration is publicly accessible via the following DOI: https://doi.org/10.17605/OSF.IO/MJGN6 (accessed on 25 March 2025) (Registration No. 10.17605/OSF.IO/MJGN6).

### 2.2. Eligibility Criteria for Study Inclusion

Studies were included if they met all eligibility criteria simultaneously. These were applied concomitantly based on the PICO framework, a widely used approach for formulating research questions in evidence-based practice. The PICO framework consists of four key components: participants (P), intervention (I), comparison (C), and outcomes (O), which guide the systematic selection of relevant studies.

(1) Participants: Older adults aged 60 years or older living in their own homes, not in hospitals or institutional settings (studies including participants with a mean age of 60 years or older were also eligible). Both healthy older adults and those with health conditions were included.
(2) Intervention: Studies implementing home modifications for older adults in their own residences, rather than in hospitals or institutions. Home modifications included structural changes (e.g., door widening, ramp installation, and improved accessibility) and the installation of assistive devices inside or outside the home (e.g., grab bars, handrails, and elevators) using either low-technology or high-technology approaches.
(3) Comparison: Studies that described the content and methods of home modifications and methods of measuring effectiveness. Studies without comparison groups were also included.
(4) Outcomes: Studies examining the content and methods of home modifications for aging in place, effectiveness assessments before and after implementation, and their impact on various outcomes, including fall risk reduction, caregiving needs and burden, functional independence (activities of daily living), occupational participation, and life satisfaction (quality of life). The effectiveness and association between home modifications and aging in place were also evaluated.

The primary inclusion criteria were based on published studies discussing home modification interventions provided in community settings. Studies were included if they met the following definition of home modifications:

Home modifications refer to changes in the residential environment to help individuals live more independently and safely in their own homes while reducing the risk of injury for both care recipients and caregivers. Home modifications include structural changes (e.g., door widening, ramp installation, and improved accessibility) and the installation of assistive devices inside and outside the home (e.g., grab bars, handrails, and elevators).

The exclusion criteria were as follows:

(1) Studies focusing on hospital and institutional environmental modifications.
(2) Studies that targeted individuals with functional disabilities but did not specifically focus on older adults.
(3) Studies that implemented home modifications but did not evaluate their effectiveness.
(4) Studies deemed irrelevant or duplicate studies.
(5) Studies without full-text availability or only available in abstract form.
(6) Review articles, letters, study protocols, poster presentations, and non-original articles such as books.
(7) Conference presentations, academic conference abstracts, and dissertations.

### 2.3. Search Strategy

Following the *Cochrane Handbook for Systematic Reviews of Interventions*, a comprehensive search was conducted in three online databases—Medline, Embase, and the Cochrane Central Register of Controlled Trials (CENTRAL)—for relevant publications between 1 January 2010 and 16 July 2024. Two keyword clusters were applied to ensure comprehensive coverage of relevant studies (Table 1).

### 2.4. Study Selection

A researcher and an external expert conducted the study selection, data extraction, and quality assessment. Two reviewers independently evaluated each selected study according to the search procedure. Initially, studies were screened based on their titles and abstracts following the inclusion criteria outlined in the PICO section. Disagreements regarding study inclusion were resolved through consensus. The two reviewers then assessed the full texts of the included studies to determine final eligibility for the review. Any disagreements about inclusion were again resolved through discussion. Additionally, an external expert independently reviewed all stages of the study selection process to minimize researcher bias and enhance the reliability of the process. 

### 2.5. Data Extraction Process

First, two reviewers evaluated the relevance of each study to the research question and objectives based on the title, abstract, and keyword information and conducted data analysis accordingly. The following data were extracted from each study and recorded in a Microsoft Excel spreadsheet: title, author(s), publication year, journal, study country, study design, type of home modifications, participant characteristics, assessment tools, outcomes, and effectiveness.

This systematic review analyzed various aspects of home modification interventions supporting aging in place for older adults. After assessing the general characteristics of the included studies, key research themes such as fall prevention effects, environmental modifications for older adults with cognitive decline, and the impact of home modifications on quality of life were classified to structure the study findings. Thematic analysis and content analysis methodologies were applied to categorize study topics, compare and organize similarities and differences across studies, and interpret the findings using a narrative synthesis approach, considering heterogeneity among the studies. The results were presented in tabular format for clarity.

### 2.6. Study Quality Assessment

A researcher and an external expert conducted a qualitative assessment of 20 selected studies. The Physiotherapy Evidence Database (PEDro) scale and the Methodological Index for Nonrandomized Studies (MINORS) were used to evaluate the methodological quality of the selected studies. The PEDro scale was applied to assess randomized controlled trials (RCTs), while MINORS was used for non-randomized controlled studies. Two independent reviewers used these tools to evaluate the level of evidence in each study. In cases where the reviewers’ opinions differed, consensus was reached through discussion to determine the final quality score.

The PEDro scale is a validated tool for assessing the methodological quality of RCTs, using a binary response system (‘Yes’ or ‘No’), with a maximum score of 10 points (number of ‘Yes’ responses). Based on methodological criteria, the quality assessment was categorized as follows: scores of 9–10 were rated as ‘excellent’, 6–8 as ‘good’, 4–5 as ‘fair’, and below 4 as ‘poor’ [26].

For non-randomized controlled studies, the MINORS score was used. The maximum ideal score for non-comparative studies was 16, whereas for comparative studies, the maximum was 24 [27]. The items are scored 0 (not reported), 1 (reported but inadequate), or 2 (reported and adequate). The global ideal score is 16 for non-comparative studies and 24 for comparative studies. The methodological quality percentage was then calculated and classified as follows: less than 25% was considered ‘very low methodological quality’, 25–49% as ‘low quality’, 50–74% as ‘moderate quality’, and 75% or higher as ‘high quality’. This classification approach, using continuous criteria, has been reported in previous research [28].

## 3. Results

### 3.1. Literature Search and Selection

**Figure 1:**
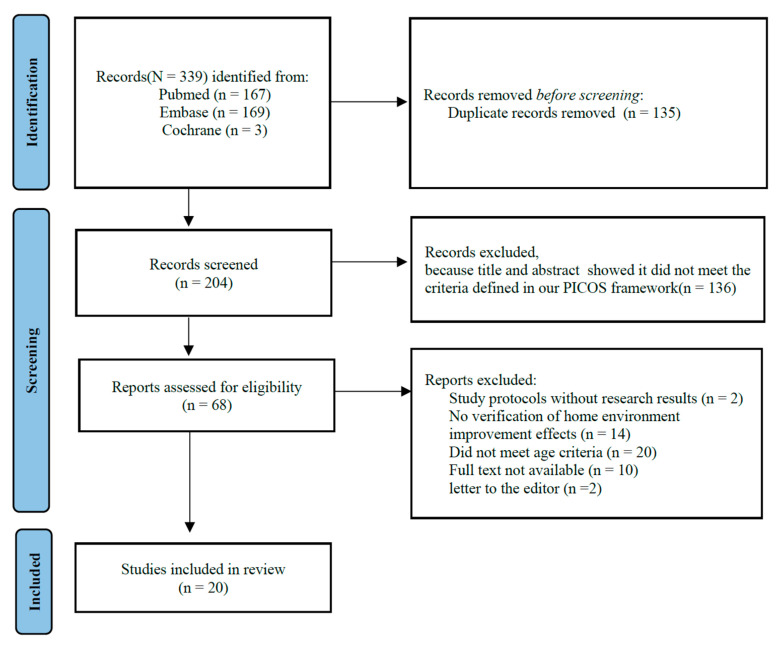
PRISMA flowchart shows details of the processes related to the identification, screening, and selection processes. PICO = participants, interventions, comparisons, outcomes study design.

A total of 339 studies were initially identified through the search process. After removing 135 duplicate studies, the remaining studies were assessed based on the inclusion criteria. As a result, 136 studies were excluded. A full-text evaluation was conducted on 68 studies, after which an additional 48 studies were excluded. Consequently, 20 studies were finally included in this systematic review (Figure 1).

### 3.2. Study Quality Assessment

The PEDro scale was applied to five randomized controlled trials (RCTs), while the MINORS scale was used to assess 15 non-randomized controlled trials (NCTs). The PEDro scale scores for all five RCTs were 9 points, indicating an ‘Excellent’ quality rating (Table 2). The MINORS scale scores for 14 studies, based on a maximum score of 16, ranged from 6 points (‘Low’) to 14 points (‘High’), while one study, evaluated based on a maximum score of 24, received a score of 23 points (‘High’) (Table 3).

### 3.3. General Characteristics of Studies

The general characteristics of the studies were summarized based on the author, year, title, journal name, study location, study design, target group, sample size, mean participant age, and gender distribution. The data in Table 4 are presented in descending order, prioritizing the most recent publications.

Among the 20 studies analyzed, 12 (60%) were conducted within the past five years, with the United States representing the largest proportion (five studies, 25%). Regarding study design, there were five randomized controlled trials (25%) and 15 non-randomized controlled trials (75%). The target population primarily included older adults aged 60 and older, accounting for 12 studies (60%), including individuals with a history of falls. The remaining studies focused on individuals with functional impairments, such as those with Parkinson’s disease, dementia, cognitive decline, or disabilities. In terms of gender distribution, 17 studies (85%) reported a higher proportion of male participants, while one study (5%) had more female participants. The remaining two studies (10%) either did not specify gender distribution or deemed it inapplicable. Participants’ ages ranged from their 60s to 80s, reflecting a diverse older adult population.

### 3.4. Intervention Type, Outcome Measurements, and Main Results of Studies

Table 5 summarizes the intervention types, session frequency and duration, outcome measurement tools, key findings, and the overall effectiveness of home modifications. All studies examined home modification interventions and their impact. Three studies [22,34,36] indirectly assessed home modifications’ role in improving residential environments. A total of 15 studies lacked a control group. Eight studies [14,22,34,37,39,42,43,44] conducted a single evaluation and data collection session, while most studies implemented home modifications following an initial assessment and conducted follow-up evaluations. The number of sessions primarily referred to home visits for evaluation. Five studies [21,32,33,40,41] included four sessions, another five studies [29,30,31,35,38] included three sessions, one study [36] had two sessions, and one study [45] included five sessions. The study with five sessions conducted 90 min home visits, providing both interventions and education. Evaluations were generally scheduled weekly, monthly, or every two to three months.

Regarding outcome measurement tools, the Falls Efficacy Scale, assessing fear of falling, was used in four studies [21,30,35,41], while fall incidence was reported in two studies [32,44]. The Housing Enabler (HE), measuring residential accessibility, was used in two studies [14,36], and the Katz ADL, evaluating functional independence, was employed in two studies [38,40]. The EuroQol Questionnaire, assessing the quality of life, appeared in two studies [33,40]. The Westmead Home Safety Assessment (WeHSA) was used in one study [21]. Additional tools were used to measure independent living, fall risk, home safety, functional mobility, and balance.

Key findings indicated that fall reduction was reported in seven studies [21,30,32,35,38,44,45], while six studies [14,29,30,33,43,45] observed improved mobility. Increased functional independence was reported in four studies [31,42,44,45], and quality-of-life enhancement in four studies [30,31,40,44]. A decrease in fear of falling was noted in four studies [29,32,35,40]. Other findings included improved adherence to home modifications, enhanced accessibility after barrier removal, reduced home safety risks, decreased anxiety and depression, shorter caregiving time, and greater self-efficacy.

Regarding effectiveness, studies were classified as “Effective” if statistically significant improvements were observed across all measured outcomes. Studies where some outcomes showed statistical significance but lacked consistent overall results or had methodological limitations were categorized as “Correlation Identified”. Among the 20 studies, 13 (65%) [21,29,30,32,33,35,37,38,39,40,42,44,45] found home modifications to be effective, contributing to fall reduction, improved functional independence, and better quality of life. Notably, bathroom modifications, grab bars, and stair railings were identified as the most impactful interventions. Additionally, reductions in emergency hospitalizations and caregiver burden were reported following home modifications.

Meanwhile, seven studies (35%) [14,22,31,34,36,41,43] found that home modifications were strongly associated with residential accessibility and lifestyle factors. These studies suggested that effectiveness varied based on socioeconomic status and living environment. Some reported that older adults in need of home modifications did not actively receive interventions. While home modifications improved functional independence and quality of life, some studies also noted a potential increase in caregiver burden as a negative consequence.

### 3.5. Home Modification Details of Studies

Table 6 summarizes the home modifications analyzed in 20 studies. Mobility and accessibility improvements and bathroom safety enhancements were implemented in all studies (100%), including threshold removal, doorway widening, stair lift installation, grab bars, and non-slip mats. Fall prevention measures were applied in 18 studies (90%), incorporating non-slip flooring, lighting improvements, and mobility training.

Kitchen and living space modifications were present in 15 studies (75%), while support for independent living was included in 14 studies (70%), involving cooking area adjustments and assistive device provision. Stair safety enhancements (12 studies, 60%) and lighting improvements (10 studies, 50%) were also emphasized. In contrast, hazard removal (eight studies, 40%) and outdoor environment modifications (seven studies, 35%) were reported less frequently.

### 3.6. A Multidimensional Analysis of Home Modification for Aging in Place Among Older Adults

This study provides a comprehensive analysis of the impact of home modifications on aging in place among older adults (Table 7). The findings include both statistically significant results and notable trends, even when statistical significance was not observed. The analysis examined the effects of home modifications on fall prevention (12 studies), activities of daily living (ADL) improvement (six studies), quality-of-life enhancement (five studies), and home safety improvements (four studies). Additional areas of research included housing accessibility (two studies), modifications for older adults with cognitive decline (two studies), caregiver burden reduction (two studies), cost-effectiveness analysis (two studies), and socioeconomic disparities (two studies).

Notably, fall prevention and increased independence were the most prominent effects, while housing accessibility and tailored interventions contributed to improved quality of life. Home modifications also reduced caregiver burden, emphasizing the need for economic and policy support. These findings highlight the importance of continued research and policy initiatives to promote independent living and a safe home environment for older adults.

### 3.7. Integrated Analysis of Health Changes and Home Modification Among Older Adults

This study analyzed the effectiveness of home modifications from three perspectives: age-related physical changes, the maintenance of physical function and balance, and cognitive function changes (Table 8).

First, home modifications effectively prevented falls, maintained functional independence, and improved quality of life. Interventions such as grab bars, non-slip mats, and stair railings significantly reduced fall incidence, particularly in high-risk areas like bathrooms. Enhanced home accessibility improved mobility and increased the likelihood of aging in place.

Second, combining home modifications with exercise was the most effective strategy for maintaining physical function and balance. These interventions improved mobility, reduced fear of falling, and enhanced physical independence. Notably, grab bars and stair railings played a crucial role in stability and movement.

Third, for older adults with cognitive impairment, home modifications that maintained familiar environments while enhancing safety were essential. Gradual adjustments, rather than abrupt changes, supported memory retention and spatial awareness.

Additionally, home modifications contributed to cost savings by preventing falls, with the greatest cost-effectiveness observed in high-risk groups. However, some studies noted an increase in caregiver burden, highlighting the need for integrated support measures.

In conclusion, systematic and tailored home modifications addressing physical, functional, and cognitive changes are essential for fall prevention and independent living. These findings emphasize the necessity of a multidimensional intervention strategy.

## 4. Discussion

Previous studies have primarily examined the individual effects of home modifications, focusing on fall prevention, physical function maintenance, and accessibility improvements. However, this study goes beyond physical safety, analyzing home modifications as a key factor in promoting aging in place from a multidimensional perspective. Specifically, it considers the impact of technological advancements and policy support on the effectiveness and accessibility of home modifications, emphasizing the need for practical and sustainable intervention strategies. Through this integrated approach, the study explores the broader impact on older adults’ well-being and, based on these findings, proposes theoretical implications, practical applications, and future research directions.

### 4.1. Methodological Considerations

This study conducted a systematic literature review following PRISMA 2020 guidelines to examine the multidimensional effects of home modifications in promoting aging in place for older adults. To enhance study reliability, clear inclusion and exclusion criteria were established. Methodological quality was assessed using the PEDro and MINORS scales: PEDro for randomized controlled trials (RCTs) [47] and MINORS for non-randomized studies. However, due to the nature of home modifications, blinding participants and intervention providers was inherently challenging in RCTs, leading to methodological limitations. Many studies also received low MINORS scores on prospective data collection, objective outcome evaluation, follow-up periods, and sample size estimation. Future research should incorporate independent evaluators, objective assessment tools, and adequate follow-up periods to address these limitations.

This study reviewed research from 2010 to 2024 to reflect advancements in home modification policies, assistive technologies, and interventions. However, focusing on recent studies may introduce publication bias, as studies with significant positive effects are more likely to be published. To mitigate this, future systematic reviews should include gray literature, such as government reports, conference proceedings, and unpublished studies [48].

Significant heterogeneity was observed across studies in intervention methods, assessment tools, and participant characteristics, limiting meta-analysis feasibility. Assessment methods ranged from self-reports and clinician evaluations to biomechanical measurements, reducing comparability and generalizability. Future research should adopt standardized assessment tools, such as the Housing Enabler [49] or Westmead Home Safety Assessment (WeHSA) [50], and apply statistical measures like I^2^ statistics and subgroup analyses to quantify heterogeneity [51,52]. A key limitation was the lack of long-term follow-up data, making it difficult to assess the sustained effects of home modifications. Future studies should establish appropriate follow-up periods, employ objective outcome measures, and use bias assessment tools [53], such as ROBINS-I [54], to enhance reliability. Sensitivity analyses [51] will further strengthen the findings’ validity. By addressing these methodological limitations and proposing specific improvements, future research can provide more robust, evidence-based home modification strategies to effectively support aging in place for older adults.

### 4.2. Primary Types and Specific Applications of Home Modifications for Older Adults

This study analyzed 20 studies to identify specific home modifications aimed at improving mobility, safety, and independence among older adults. The findings revealed that mobility and accessibility improvements (100%) and bathroom safety enhancements (100%) were the most common modifications, playing a crucial role in fall prevention and safe indoor movement. The most frequently implemented fall prevention strategies (90%) included non-slip flooring, stair handrails, and improved lighting.

Kitchen and living space modifications (75%) primarily focused on adjusting cabinet heights and optimizing cooking areas. Independent living support interventions (70%) involved adjustable beds, assistive devices, and caregiver education programs. Additionally, stair safety enhancements (60%) and lighting improvements (50%) were widely adopted, with an increasing shift toward LED lighting and sensor-based automation. However, hazard removal (40%) and outdoor modifications (35%) were implemented less frequently, suggesting the need for further research and policy support. Future studies should prioritize outdoor accessibility improvements, including ramps, threshold removal, doorway widening, and slip-resistant walkways [21].

These findings underscore that home modifications extend beyond structural changes, representing a multidimensional intervention supporting mobility, safety, and independent living. The widespread adoption of mobility and bathroom modifications highlights their fundamental importance. Additionally, the growing emphasis on fall prevention, lighting improvements, and smart technologies reflects an increasing shift toward technology-driven solutions. The trend toward personalized home modifications has led to the adoption of assistive devices, caregiver education, and smart home technologies, such as automated lighting, motorized blinds, and IoT-based home systems.

Despite the growing recognition of smart home technology, research on its effectiveness and evaluation methodologies remains limited [55]. Future studies should adopt systematic evaluation frameworks to assess its impact. The Technology Acceptance Model (TAM) can quantify user-friendliness, ease of use, and perceived usefulness of smart home systems [51]. Smart-sensor-based fall prevention should be evaluated using objective metrics, such as fall incidence, severity, and emergency response time [56]. The cost-effectiveness and sustainability of IoT-based smart homes should be analyzed through installation costs, maintenance expenses, and long-term viability [57]. Lastly, user satisfaction and real-world impact should be assessed through surveys and in-depth interviews to provide qualitative insights [56]. By applying these systematic methodologies, future research can generate empirical evidence, advancing a clearer, more structured understanding of smart home technologies in supporting aging in place.

### 4.3. The Multidimensional Effects of Home Modifications on Aging in Place Among Older Adults

In this study, a multidimensional analysis of home modifications aimed at supporting aging in place among older adults was conducted, examining their effects on fall prevention, functional independence, quality of life, household stability, environmental safety, accessibility, cognitive decline, caregiver burden, cost-effectiveness, and socioeconomic disparities. Home modifications are an effective intervention for fall prevention, with even greater benefits when combined with exercise programs. A systematic review by Stark et al. [56] found that home modifications significantly reduced fall risk and increased activity participation among older adults with physical impairments. Similarly, Chase et al. [58] reported that older adults who participated in both home modifications and regular exercise programs had a significantly lower incidence of falls compared to those receiving only home modifications. These findings confirm the complementary role of home modifications and exercise in maximizing fall prevention outcomes, emphasizing the need for continuous assessment and individualized adjustments based on functional changes.

Home modifications also contribute to improved quality of life, independence, and psychological stability. However, some studies indicate limitations, including a slight increase in caregiver burden. While modifications improve mobility and social participation, they may also place additional demands on caregivers [59]. Certain studies suggest that home modifications alone may not always be sufficient and are most effective when tailored to individual living environments and functional abilities [60]. Therefore, a personalized home modification strategy that considers functional capacity, cognitive status, and lifestyle is essential. Additionally, technology-driven interventions, such as smart home systems, may further enhance effectiveness. These findings highlight the need for meticulously designed home modifications to promote safe and independent living, along with policies supporting personalized interventions.

For older adults with cognitive impairments, home modifications have been effective in reducing falls and improving functional abilities, though they have also been associated with a slight increase in caregiver burden. This may be due to the challenges older adults with cognitive impairments face in adapting to environmental changes, requiring caregivers to provide continuous monitoring and support after modifications. To address this, gradual adjustments rather than abrupt changes should be implemented, along with caregiver education and expert involvement [61]. While home modifications can reduce caregiver burden and promote independence, sudden environmental changes may cause confusion among those with cognitive impairments. Caregivers often struggle to anticipate their full impact, potentially increasing their burden. Therefore, a gradual approach, caregiver education, and expert intervention are critical to maximizing effectiveness [62]. Generally, home modifications have also proven to be effective in reducing caregiving time and burden, with bathroom modifications being one of the most widely implemented interventions. However, as older adults adapt to modified environments, the role of caregivers may expand, requiring complementary support measures. A multifaceted approach, including gradual adjustments and caregiver education, should be integrated into home modification planning. Additionally, long-term policy support is essential to reduce the overall caregiver burden [63,64].

Home modifications are a cost-effective intervention, particularly for high-risk populations. Low-cost modifications, such as grab bars and improved lighting, are highly cost-effective, while full-scale renovations, though beneficial in the long term, require substantial initial investment. However, research on the cost-effectiveness of home modifications remains limited. Future studies should conduct comprehensive cost-effectiveness analyses comparing low-cost vs. high-cost modifications and traditional home modifications vs. smart-home-technology-based interventions to optimize public funding allocation.

Access to home modification programs varies based on socioeconomic factors such as residential location, disability status, and income level. Research has highlighted that socioeconomic disparities may exclude low-income and rural older adults from receiving home modification services. Key policy initiatives addressing this issue include the Medicaid Waiver Program, Community Development Block Grant (CDBG), USDA Section 504 Program (U.S), Japan’s Long-Term Care Insurance Act, and the UK’s Disabled Facilities Grant (DFG) and Home Improvement Agencies (HIAs) model [65,66]. Future research should conduct a comparative analysis of successful policy models from the U.S., Japan, and the UK to establish a more defined strategic policy direction for supporting aging in place, tailored to the specific circumstances of each country. Additionally, standardized protocols should be developed for assessing and implementing home modifications, and interdisciplinary collaboration among occupational therapists, architects, and social workers should be promoted to enhance professional capacity and practical implementation.

### 4.4. The Interactions and Comprehensive Impacts of Health Changes and Home Modifications Among Older Adults

This study comprehensively analyzed the effects of home modifications for older adults from three key perspectives: physical aging changes, maintenance of physical function and balance, and cognitive function changes. These factors are interconnected rather than independent, requiring an integrated approach to maximize their benefits. The interaction between age-related physical changes, such as muscle weakness, balance impairments, functional decline, and environmental modifications necessitates continuous and systematic interventions. As aging progresses, muscle weakness and balance impairments become major contributors to falls [67]. Studies indicate that home modifications, including handrail installation and optimized movement pathways, effectively mitigate physical function decline [68]. Systematic home modification programs have been shown to significantly reduce fall incidence rates [69]. Since home modifications play a critical role in compensating for functional decline, interventions should not be one-time measures but should involve regular monitoring and adjustments. As older adults’ physical needs evolve, continuous environmental assessments and modifications should be conducted. A customized approach that considers an individual’s lifestyle and residential environments is essential.

To maintain physical function and balance, a combined intervention of home modifications and exercise is necessary. Age-related musculoskeletal changes impact mobility and balance control [70]. Therefore, integrating balance and strength training exercises with home modifications enhances mobility and functional independence in older adults [71]. Research shows that installing stair handrails and non-slip flooring significantly improves balance, with even greater benefits observed when paired with structured exercise programs [72]. The integration of home modifications and exercise is crucial because it directly impacts physical function maintenance. Environmental modifications alone are insufficient to fully support mobility in older adults; a targeted exercise regimen focusing on balance and strength must be included. Regular balance and strength training is a highly effective strategy for improving mobility and autonomy in older adults.

For older adults with cognitive impairments, home modifications should preserve a familiar environment while enhancing safety to prevent falls and behavioral disturbances and to promote functional independence. Cognitive impairments can make adapting to environmental changes challenging, increasing fall risk. Maintaining familiar surroundings while incorporating safety measures such as handrails, improved lighting, and non-slip flooring effectively prevents falls and supports independence. Studies highlight that bathroom modifications are particularly effective in reducing fall risk [73]. An effective approach may involve starting with non-invasive adjustments, such as improved lighting, before gradually incorporating handrails or furniture rearrangement.

To achieve these objectives, customized support programs tailored to older adults’ health conditions are necessary. Additionally, collaborative efforts between governments and local communities are essential for implementing home modification policies. With these combined efforts, older adults can live more safely and independently.

## 5. Conclusions

This study systematically and comprehensively analyzed the effects of home modifications in supporting aging in place for older adults. The findings indicate that home modifications extend beyond structural changes, offering significant benefits in fall prevention, functional independence, quality of life, caregiver burden reduction, and cost-effectiveness. However, to maximize these effects, personalized interventions that consider an individual’s physical and cognitive characteristics and living environment, along with continuous maintenance and appropriate technology integration, are essential. Additionally, the impact of home modifications varies based on socioeconomic background, cognitive function, and physical condition, highlighting the need for a strategic and individualized approach.

### 5.1. Theoretical Implications

This study establishes that home modifications are not merely structural changes but a fundamental factor in promoting physical, psychological, and social well-being. Unlike previous research that primarily focused on isolated effects such as fall prevention and mobility improvement, this study integrates multiple intervention factors, analyzing the broader impact of home modifications on older adults’ overall lives. This approach addresses a gap in the literature regarding the multidimensional effects of home modifications and confirms their essential role in supporting aging in place. Additionally, the study emphasizes the need for standardized evaluation tools, such as the Housing Enabler and Westmead Home Safety Assessment, to enhance research consistency and comparability. Establishing a methodological framework through these standardized tools will enable more objective analysis of home modification effects.

### 5.2. Practical Applications

This study confirms that home modifications are a cost-effective intervention that reduces caregiver burden. However, accessibility to home modification programs remains limited for low-income and rural-dwelling older adults. To address these disparities, policymakers should reference successful financial support models, such as the Medicaid Waiver system, Community Development Block Grant (CDBG), and USDA Section 504 Rural Repair and Rehabilitation Grants in the United States, to develop more effective support systems. Additionally, the adoption of smart home technologies, such as sensor-based lighting and voice-controlled systems, should be actively promoted to enhance independence and convenience for older adults. Interdisciplinary collaboration among occupational therapists, architects, and social workers is essential for developing personalized home modification strategies that address the physical and cognitive needs of older adults. This approach should extend beyond physical modifications, creating a residential environment that supports long-term independence.

### 5.3. Future Research Directions

Future research should focus on comparative studies between smart home technology and traditional home modifications to identify the most effective intervention strategies. To evaluate the effectiveness of smart home technologies, studies should employ the Technology Acceptance Model (TAM) to assess older adults’ technology adoption rates and utilize sensor-based data to analyze fall incidence rates and severity. Additionally, a comparative cost-effectiveness analysis between smart home technologies and conventional home modifications should assess economic feasibility, considering both initial installation costs and long-term maintenance expenses. Further research should develop customized home modification models for diverse older adult populations, including those with severe physical impairments, mild dementia, and economic disadvantages, to determine the most suitable interventions for each group. Moreover, comparative policy analyses of home modification support programs in the United States, Japan, and the United Kingdom should be conducted to develop an optimal policy framework adaptable to various local communities. To achieve these goals, future research should incorporate standardized evaluation frameworks from the early stages of study design, ensuring systematic and objective analysis while prioritizing studies with high practical applicability.

This study contributes to enhancing safety and functional independence for older adults, ultimately facilitating successful aging in place. To achieve this, empirical studies must provide scientific evidence and conduct precise analyses of home modification effectiveness. With these efforts, older adults can maintain a safer and more independent lifestyle, ultimately leading to a healthier and more sustainable aging process.

## Figures and Tables

**Table 1:** Keywords related to old age and home modification.

Domain	Keywords
Old Age	“Aged” [MeSH] [Emtree] OR “Elderly” [TIAB] OR “Older People” [TIAB] OR “Older Adult” [TIAB] OR “Older Person” [TIAB] OR “Aging Population” [TIAB] OR “Geriatric” [TIAB] OR “Senior” [TIAB]
Home Modification	“Home Modification” [TIAB] OR “Home Adaptation” [TIAB] OR “Home Renovation” [TIAB] OR “Home Improvement” [TIAB] OR “Barrier-Free Remodeling” [TIAB] OR “Home Environment Adaptation” [TIAB]

**Table 2:** PEDro scale score.

	Stasi et al. (2021) [29]	Tsekoura et al. (2021) [30]	Jeon et al. (2020) [31]	Stark et al. (2017) [21]	Kamei et al. (2015) [32]
1. Eligibility criteria	Y	Y	Y	Y	Y
2. Randomization	Y	Y	Y	Y	Y
3. Hidden assignment	Y	Y	Y	Y	Y
4. Group homogenous	Y	Y	Y	Y	Y
5. Subjects blinded	N	N	N	N	N
6. Therapists blinded	N	N	N	N	N
7. Assessors blinded	Y	Y	Y	Y	Y
8. Follow-up subjects	Y	Y	Y	Y	Y
9. Intention to treat	Y	Y	Y	Y	Y
10. Comparisons between groups	Y	Y	Y	Y	Y
11. Scoring and variability measures	Y	Y	Y	Y	Y
Total score	9	9	9	9	9
Quality rating	Excellent	Excellent	Excellent	Excellent	Excellent

**Table 3:** MINORS score.

	Riera Arias et al. (2024) [33]	Kim et al. (2024) [34]	Hawkis et al. (2024) [35]	Andersson et al. (2023) [36]	Schiller et al. (2023) [37]	Yeni et al. (2022) [38]	Hollinghurst et al. (2022) [39]	Schorderet et al. (2022) [40]	Malmgren Fänge et al. (2021) [41]	Carnemolla et al. (2019) [42]	Wilson et al. (2019) [43]	Pettersson et al. (2018) [14]	Wilson et al. (2017) [44]	Somerville et al. (2016) [45]	Harvey et al. (2014) [46]
1. A clearly stated aim	2	2	2	2	2	2	2	2	2	2	2	2	2	2	2
2. Inclusion of consecutive patients	1	1	1	1	1	1	2	1	1	1	1	1	0	1	1
3. Prospective collection of data	2	0	1	2	2	2	2	2	2	2	1	0	0	2	0
4. Endpoints appropriate to the aim of the study	2	2	2	2	1	2	2	2	2	2	2	2	2	2	2
5. Unbiased assessment of the study endpoint	0	1	0	1	0	1	1	1	1	1	0	0	1	1	1
6. Follow-up period appropriate to the aim of the study	1	0	1	2	1	2	2	2	2	2	0	0	1	2	0
7. Loss to follow up less than 5%	1	0	1	1	1	2	2	2	1	1	2	2	0	2	0
8. Prospective calculation of the study size	2	0	0	1	0	2	2	0	1	1	0	0	1	0	1
Items 9–12 are only applicable to comparative studies															
9. An additional control group	-	-	-	-	-	-	2	-	-	-	-	-	-	-	-
10. Contemporary Groups	-	-	-	-	-	-	2	-	-	-	-	-	-	-	-
11. Baseline equivalence of groups	-	-	-	-	-	-	2	-	-	-	-	-	-	-	-
12. Adequate statistical analyses	-	-	-	-	-	-	2	-	-	-	-	-	-	-	-
Total score/Max. possible score	11/16	6/16	8/16	12/16	8/16	14/16	23/24	12/16	12/16	12/16	8/16	7/16	7/16	12/16	7/16
Quality rating	Moderate	Low	Moderate	Moderate	Moderate	High	High	Moderate	Moderate	Moderate	Moderate	Low	Low	Moderate	Low

**Table 4:** General characteristics of studies.

No.	Author (Year)	Title	Journal	Country of the Study	Study Design	Target Group	Sample Size	Age, M ± SD (Range)	Sex, Male/Female(%)
1	Riera Arias et al. (2024) [33]	Improving quality of life in older adults with the decline syndrome: The role of occupational therapy in primary care	*Atención Primaria*	Spain	Quasi-experimental pre–post study	Older adults with decline syndrome	EG: 62 CG: Not applicable (no control group)	83.3 ± 8.92	24 (38.7)/38 (61.3)
2	Kim et al. (2024) [34]	Exploring Differences in Home Modification Strategies According to Household Location and Occupant Disability Status: 2019 American Housing Survey Analysis	*Journal of Applied Gerontology*	USA	Cross-sectional survey study	Older adults (65+) in different household locations	EG: 3413 CG: Not applicable	Not specified (all participants aged 65+)	1355 (39.7)/2058 (60.3)
3	Hawkins et al. (2024) [35]	Evaluation of a Fall Prevention Program to Reduce Fall Risk and Fear of Falling Among Community-Dwelling Older Adults and Adults with Disabilities	*Clinical Interventions in Aging*	USA	Program evaluation study	Older adults and adults with disabilities	EG: 241 CG: Not applicable (no control group)	75	40 (18.3)/179 (81.7)
4	Andersson et al. (2023) [36]	Environmental barriers and housing accessibility problems for people with Parkinson’s disease: A three-year perspective	*Scandinavian Journal of Occupational Therapy*	Sweden	Longitudinal cohort study	People with Parkinson’s disease	EG: 138 CG: Not applicable	68.3 ± 8.6	92 (66.7)/46 (33.3)
5	Schiller et al. (2023) [37]	A Home Repair and Modification Program Embedded Within Mount Sinai Visiting Doctors	*Journal of Applied Gerontology*	USA	Pilot mixed-methods study (quantitative and qualitative analyses)	Homebound older adults in primary care	EG: 33 CG: Not applicable (no control group)	Sixty-three percent of participants were ≥70 years old (age range: <60 to 90+ years)	5 (22)/28 (78)
6	Yeni et al. (2022) [38]	Nurse-led home modification interventions for community-dwelling older adults with dementia and their impact on falls prevention	*British Journal of Healthcare Assistants*	Turkey	Quasi-experimental study	Older adults with dementia living at home	EG: 42 CG: Not applicable	79.04 ± 7.8	18 (42.9)/24 (57.1)
7	Hollinghurst et al. (2022) [39]	Do home adaptation interventions help to reduce emergency fall admissions? A national longitudinal data-linkage study of 657,536 older adults living in Wales (UK) between 2010 and 2017	*Age and Ageing*	UK (Wales)	Longitudinal cohort study	Older adults (60+) living in the community	EG: 123,729 CG: 533,807 Total: 657,536	EG: 78.03 CG: 70.6	EG: 46,648 (37.76)/77,081 (62.24) CG: 257,352 (48.21)/276,455 (51.79)
8	Schorderet et al. (2022) [40]	Needs, benefits, and issues related to home adaptation: a user-centered case series applying a mixed-methods design	*BMC Geriatrics*	Switzerland	Case series, mixed-methods study	Older adults living at home	EG: 15 homes CG: Not applicable	75.1 ± 7.3 (65–86)	4 (22.2)/14 (77.8)
9	Malmgren Fänge et al. (2021) [41]	One-Year Changes in Activities of Daily Living, Usability, Falls and Concerns about Falling, and Self-Rated Health for Different Housing Adaptation Client Profiles	*International Journal of Environmental Research and Public Health*	Sweden	Longitudinal cohort study	Older adults receiving housing adaptation (HA) grants	EG: 108 CG: Not applicable	75 ± 13	29 (27)/79 (73)
10	Stasi et al. (2021) [29]	Motor Control and Ergonomic Intervention Home-Based Program: A Pilot Trial Performed in the Framework of the Motor Control Home Ergonomics Elderlies’ Prevention of Falls (McHeELP) Project	*Cureus*	Greece	Pilot randomized controlled trial (RCT)	Community-dwelling older adults (≥65 years) with a history of falls	EG: 10 CG: 10 Total: 20	EG: 79.4 ± 5.27 CG: 76.4 ± 6.03	EG: 6 (60)/4 (40) CG: 6 (60)/4 (40)
11	Tsekoura et al. (2021) [30]	Methodology of a home-based motor control exercise and ergonomic intervention programme for community-dwelling older people: The McHeELP study	*Journal of Frailty, Sarcopenia and Falls*	Greece	Randomized controlled trial	Community-dwelling older adults with a history of falls	EG: Not specified (planned 1:1 randomization) CG: Not specified	≥65 years (inclusion criteria), no further details provided	Not specified
12	Jeon et al. (2020) [31]	Feasibility and potential effects of interdisciplinary home-based reablement program (I-HARP) for people with cognitive and functional decline: a pilot trial	*Aging & Mental Health*	Australia	Parallel-group randomized controlled pilot trial	Older adults with cognitive decline and caregivers	EG: 9 dyads CG: 9 dyads Total: 18 dyads	EG: 79 (64–85) CG: 81 (74–91)	EG: 3 (33.3)/6 (66.7) CG: 5 (55.6)/4 (44.4)
13	Carnemolla et al. (2019) [42]	Housing Design and Community Care: How Home Modifications Reduce Care Needs of Older People and People with Disability	*International Journal of Environmental Research and Public Health*	Australia	Pre–post study	Older adults and people with disabilities receiving home modifications	EG: 157 CG: Not applicable	72	72 (45.9)/85 (54.1)
14	Wilson et al. (2019) [43]	The hidden impact of home adaptations: Using a wearable camera to explore lived experiences and taken-for-granted behaviours	*Health & Social Care in the Community*	UK	Qualitative case study	Older adults with home adaptations	EG: 6 CG: Not applicable	Not provided (range: 65+)	1 (16.7)/5 (83.3)
15	Pettersson et al. (2018) [14]	Housing accessibility for senior citizens in Sweden: Estimation of the effects of targeted elimination of environmental barriers	*Scandinavian Journal of Occupational Therapy*	Sweden	Cross-sectional study	Older adults in ordinary housing	EG: 609 dwellings (370 EA study, 239 HHPD study) CG: Not applicable	EA study: 85 HHPD study: 70 (45–93)	EA study: 93 (25)/277 (75) HHPD study: not specified
16	Stark et al. (2017) [21]	Protocol for the home hazards removal program (HARP) study: a pragmatic, randomized clinical trial and implementation study	*BMC Geriatrics*	USA	Randomized clinical trial (RCT)	Older adults at risk of falls	EG: 150 CG: 150 Total: 300	77	99 (33)/201 (67)
17	Wilson et al. (2017) [44]	Home modification to reduce falls at a health district level: Modeling health gain, health inequalities and health costs	*PLOS ONE*	New Zealand	Modeling study	Older adults (65+) in a health district	EG: 42,000 (modeled population) CG: Not applicable	Not specified (65+ population)	Not specified
18	Somerville et al. (2016) [45]	Occupational Therapy Home Modification Assessment and Intervention	*American Journal of Occupational Therapy*	USA	Case report	Older adults with functional limitations	EG: 1 case CG: Not applicable	75	0 (0)/1 (100)
19	Kamei et al. (2015) [32]	Effectiveness of a home hazard modification program for reducing falls in urban community-dwelling older adults: A randomized controlled trial	*Japan Journal of Nursing Science*	Japan	Randomized controlled trial (RCT)	Community-dwelling older adults (≥65 years)	EG: 67 CG: 63 Total: 130	EG: 75.7 ± 6.7 CG: 75.8 ± 6.4	EG: 11 (16.4)/56 (83.6) CG: 9 (14.3)/54 (85.7)
20	Harvey et al. (2014) [46]	Determinants of uptake of home modifications and exercise to prevent falls in community-dwelling older people	*Australian and New Zealand Journal of Public Health*	Australia	Cross-sectional survey study	Community-dwelling older adults (65+)	EG: 5681 CG: Not applicable	Not specified (all participants aged 65+)	2233 (39.3)/3448 (60.7)

**Table 5:** Intervention type, outcome measurements, and main results of studies.

No.	Author (Year)	Type of Intervention: Experimental Group	Type of Intervention: Control Group	Session Number, Frequency, and Duration	Outcome Measurements (Assessment Tool)	Results	Correlation or Effectiveness in Home Modification
1	Riera Arias et al. (2024) [33]	Occupational therapy with independence training, mobility support, and home adaptation, plus caregiver training	Not applicable (no control group)	After the initial assessment, 4 sessions over 4 weeks (1 per week)	Independence in daily activities: Barthel Index, Lawton Scale Quality of life: EuroQol Questionnaire (EQ-5D) Home suitability: Home Suitability Assessment Questionnaire	· Statistically significant: Autonomy ↑ (*p* = 0.003) Mobility ↑ (*p* = 0.001) Home adaptation ↑ (*p* < 0.001) Anxiety/depression ↓ (*p* = 0.028) Health score ↑ (*p* < 0.001) · Not statistically tested but notable: Home adaptation rate ↑ (45.2% → 79.0%)	Effective Occupational therapy improved autonomy, mobility, and home adaptation, enhancing quality of life. Home modifications were key for independence.
2	Kim et al. (2024) [34]	Home modifications from 2019 AHS (American Housing Survey) data: flooring, bathrooms, doors/windows, and driveways	Not applicable (no control group)	One-time data analysis based on the 2019 survey results	Home modifications: Self-reported in the 2019 American Housing Survey (AHS) Disability status: AHS classification of household members’ physical limitations Home location: Urban vs. rural classification in AHS	· Statistically significant: Rural older adults modified homes less and spent less (*p* < 0.001). Disabled households used more home equity loans for modifications (*p* < 0.0001). Rural areas focused on outdoor modifications and urban areas on indoor modifications (*p* < 0.05). · Not statistically tested but notable: Common modifications: flooring, bathrooms, and doors/windows. Seventy-seven percent of modifications were self-funded.	Correlation identified Home modifications varied by location and disability. Older adults in rural areas and those with disabilities modified less and spent less.
3	Hawkins et al. (2024) [35]	Safe at Home (SAH) program with home modifications (grab bars, safety railings, stair lifts, and bathtub cutouts)	Not applicable (no control group)	Three sessions, one per month, over 5–6 weeks	Safety hazards: Safety Assessment of Function and the Environment for Rehabilitation in the Home (SAFER HOME) Fear of falling: Falls Efficacy Scale (FES) Fall risk: Falls Risk of Older People in the Community (FROP-Com)	· Statistically significant: Seventy-nine percent reported no falls post-modification. Fear of falling ↓ (FES: T(107) = 5.14, *p* < 0.001). Falls per year ↓ (2.53 → 1.5, z = 5.35, *p* < 0.01). · Not statistically tested but notable: Ninety percent of older adults and eighty-six percent of disabled adults were satisfied. Common modifications: grab bars, chair lifts, shower chairs, and toilet seat adjustments.	Effective Home modifications significantly reduced falls and fear of falling. High program satisfaction reported.
4	Andersson et al. (2023) [36]	Home accessibility assessment using the Housing Enabler, identifying key barriers.	Not applicable (no control group)	Two sessions: baseline assessment and follow-up after 3 years	Housing accessibility problems: Housing Enabler (HE) Functional limitations and mobility aid dependency: Observational assessment Disease severity: Hoehn and Yahr scale (HY) Motor symptoms: Unified Parkinson’s Disease Rating Scale Part III (UPDRS III) Cognitive function: Montreal Cognitive Assessment (MoCA) Activities of daily living difficulties: Parkinson’s Disease Activities of Daily Living Scale (PADLS)	· Statistically significant: Hygiene area grab bar issues ↓ (*p* = 0.041). Stairs as the only route ↓ (*p* = 0.002). Difficulty reaching refuse bins ↑ (*p* < 0.001). Limited space around storage units ↑ (*p* < 0.001). · Not statistically tested but notable: Top 10 barriers remained but changed in ranking. Exterior barriers increased over time. Kitchens, bathrooms, and entrances remained key problem areas.	Correlation identified Home accessibility changes over time for Parkinson’s patients. Grab bars and stair alternatives helped, but new outdoor barriers emerged. Ongoing adaptations are needed.
5	Schiller et al. (2023) [37]	Home modification program within home-based primary care (HBPC)	Not applicable (no control group)	One-time assessment (August 2019–December 2020), with a second visit for home modification if needed (1–2 months)	Home safety: Standardized home assessment tool Program feasibility: Provider feedback on implementation	· Statistically significant: Home safety hazards ↓, patient satisfaction ↑ · Not statistically tested but notable: Cost per patient: USD 528. Common modifications: self-care, sleep, safety, maintenance, and mobility. Feasible and beneficial, but implementation challenges exist.	Effective Improved home safety, comfort, and independence. Feasible but needs better occupational therapy integration.
6	Yeni et al. (2022) [38]	Nurse-led home safety modifications, including grab bars, anti-slip flooring, and furniture adjustments	Not applicable (no control group)	Three sessions, one every 3 months, over 6 months	Fall risk: DENN Fall Risk Assessment Scale Home environment safety: Home Environment Risk Factors for Falls Assessment Form Activities of daily living: Katz Index of Independence in Activities of Daily Living (Katz ADL) Instrumental activities of daily living: Brody–Lawton Instrumental Activities of Daily Living (Brody–Lawton IADL)	· Statistically significant: Falls ↓ in the second 3-month period (*p* = 0.002). Forty-three percent of families modified homes; no falls in modified homes (*p* = 0.000). · Not statistically tested but notable: Most falls occurred in bathrooms and toilets. Common modifications: grab bars, anti-slip flooring, and improved lighting.	Effective Nurse-led home modifications significantly reduced falls in older adults with dementia.
7	Hollinghurst et al. (2022) [39]	Home adaptations: grab rails, stair rails, ramps, and heating improvements	Usual care without structured home modifications	Analysis of seven years of data from 2010 to 2017	Fall-related emergency admissions: Hospital records Frailty status: Electronic Frailty Index (eFI) Socioeconomic status: Welsh Index of Multiple Deprivation (WIMD)	· Statistically significant: Fall odds ↓ 3% per quarter (OR = 0.97, *p* < 0.001). Frailty and deprivation ↑ fall risk (moderate frailty OR = 2.31, severe frailty OR = 3.05). · Not statistically tested but notable: Most falls occurred in bathrooms and bedrooms. Regional differences affected intervention effectiveness.	Effective Home adaptations reduced fall-related emergency admissions over time, particularly for individuals with prior falls.
8	Schorderet et al. (2022) [40]	Individualized home adaptations, including bathroom modifications, improved lighting, and accessibility adjustments	Not applicable (no control group)	Four sessions (two pre-assessments, two post-assessments). Pre-assessments: one every 2–4 weeks. Post-assessments: at 1–2 months and 6 months. Total duration: 6–7 months.	Quality of life: EuroQol-5D-3L (EQ-5D-3L) Fear of falling: Falls Efficacy Scale International (FES-I) Functional independence: Katz Index of Independence in Activities of Daily Living (Katz ADL) Perceived difficulty in daily activities: Visual Analog Scale (VAS)	· Statistically significant: Bathroom-related difficulties ↓ 93.4%. Quality of life ↑ 9.8% (SD = 27.6). Fear of falling ↓ 12.5% (SD = 9.7). · Not statistically tested but notable: Common modifications: bathroom adaptations, shower conversions, and improved lighting. Participants reported increased safety, ease of use, and comfort.	Effective Home adaptations improved safety, independence, and quality of life. Participants experienced reduced difficulties in daily activities and lower fear of falling.
9	Malmgren Fänge et al. (2021) [41]	Home adaptations, including grab bars, threshold removal, and kitchen/bathroom modifications	Not applicable (no control group)	Four sessions (one baseline assessment, three evaluations every 3 months), one every 3 months, over 12 months	Activities of daily living (ADL): ADL Staircase Usability in home: Usability in My Home (UIMH) Concerns about falling: Falls Efficacy Scale International (FES-I) Self-rated health: EuroQoL 5D Visual Analogue Scale (EQ-VAS) Fall history: Self-reported six-month fall recall	· Statistically significant: ADL dependence ↑ in some, ↓ in others (varied *p*-values). Fear of falling ↓ in some, ↑ in others. Usability ↑ for self-care, ↓ for outdoor use. · Not statistically tested but notable: Adaptation effects varied, requiring personalized follow-ups. Cognitive impairment linked to highest functional decline risk.	Correlation identified Home adaptations had mixed results. ADLs and usability improved for some, but fall concerns and self-rated health varied. Ongoing monitoring is needed.
10	Stasi et al. (2021) [29]	Twelve-week home-based motor control exercise program combined with ergonomic home modifications	Usual care with no exercise intervention but received general home safety recommendations	Three sessions, one research team visit every 4 weeks (three self-exercise sessions per week), over 12 weeks	Functional mobility: Timed Up and Go test (TUG) Balance: Tandem Stance test Fall risk: Home Falls and Accidents Screening Tool (HOMEFAST) Quality of life: EuroQoL-5D-5L (EQ-5D-5L) Functional independence: Lower Extremity Functional Scale (LEFS) Fear of falling: Falls Self-Efficacy International Scale (FES-I)	· Statistically significant: Mobility ↑ (TUG test, *p* < 0.001). Balance ↑ (Tandem stance, *p* < 0.001). Quality of life ↑ (EQ-5D-5L VAS, *p* < 0.001). Fear of falling ↓ (FES-I, *p* = 0.001). · Not statistically tested but notable: One hundred percent program adherence. Increased confidence in mobility and home safety.	Effective McHeELP significantly improved functional mobility, balance, and quality of life while reducing fall risk.
11	Tsekoura et al. (2021) [30]	Home-based motor control exercises and ergonomic home modifications	Not applicable (no control group)	Three sessions (baseline, weeks 4–6, week 12), one visit per session (three times per week, individual exercise over 12 weeks), total 12 weeks	Functional mobility: Timed Up and Go test (TUG) Balance: Tandem stance test, Functional Reach Test (FRT) Fear of falling: Falls Efficacy Scale International (FES-I) Quality of life: EuroQol 5D (EQ-5D) Home safety: Home Falls and Accidents Screening Tool (HOMEFAST)	· Statistically significant: Falls ↓ post-intervention. Mobility ↑ (TUG test improved). Quality of life ↑ (EQ-5D improved). · Not statistically tested but notable: Common modifications: grab bars, lighting, and stair railings. Participants felt more confident and independent.	Effective Home modifications combined with motor control exercises significantly reduced falls and improved functional mobility.
12	Jeon et al. (2020) [31]	Home-based reablement (OT, RN, Neuropsychologist) + USD 1000 for home modifications	Usual care + educational materials and movie vouchers	Three sessions: initial assessment, home modification over 4 months, first evaluation at 4 months, final evaluation at 12 months	Functional independence: Disability Assessment for Dementia (DAD) Physical function and disability: Late Life Function and Disability Instrument (LLFDI-CAT) Depression: Geriatric Depression Scale (GDS-15) Quality of life: EuroQol-5D-3L (EQ5D-3L) Caregiver burden: Zarit Burden Inventory (ZBI)	· Statistically significant: Functional independence (↑ in EG, ↓ in CG, *p* < 0.05). Quality of life (↑ in EG, ↓ in CG, *p* < 0.05). · Not statistically tested but notable: Three CG participants moved to care homes, none in EG. Caregiver burden slightly increased in EG.	Correlation identified Improved function and quality of life, but results had uncertainty.
13	Carnemolla et al. (2019) [42]	Home modifications including structural improvements to accessibility and safety	Not applicable (no control group)	One session: home modifications completed in 6 months, followed by care need assessments	Care hours: Self-reported weekly hours of informal and formal care before and after modification	· Statistically significant: Weekly care hours ↓ 42% post-modification. Informal care ↓ 46%, formal care ↓ 16%. · Not statistically tested but notable: Bathroom modifications most common. Increased independence and reduced caregiver burden.	Effective Home modifications reduced caregiving needs: weekly care hours ↓ 42%, informal care ↓ 46%, formal care ↓ 16%.
14	Wilson et al. (2019) [43]	Home adaptations (ramps, stair lifts, grab bars, and bathroom modifications)	Not applicable (no control group)	One-time assessment	Daily activity patterns: Wearable camera analysis Perceived vs. actual use: Participant interviews Environmental interaction: Behavioral coding	· Not statistically tested but notable: Participants underestimated their reliance on home adaptations. Safety and mobility improved, but home attachment shaped perceptions. Participants unconsciously adapted their behaviors to the environment.	Correlation identified Home adaptations influenced daily routines, increased safety, and improved mobility. However, users often under-reported their reliance on modifications.
15	Pettersson et al. (2018) [14]	Simulated removal of five environmental barriers: thresholds, grab bars, ramps, shower stall curbs, and bathtubs.	Not applicable (no control group)	One-time simulation analysis	Housing accessibility: Housing Enabler (HE)	· Statistically significant: Accessibility ↑ in older housing and single-family homes. Removing barriers reduced accessibility issues by up to 35%. · Not statistically tested but notable: Fifty percent of homes lacked grab bars in hygiene areas. Eighty percent of single-family houses had indoor steps/thresholds. Home modifications could benefit 40–82% of older adults.	Correlation identified Targeted modifications improved accessibility for individuals with limitations. Systematic home modifications can enhance aging in place.
16	Stark et al. (2017) [21]	Home hazard removal by occupational therapists, including grab bars, slip-resistant flooring, lighting, and environmental modifications	Usual care with general aging services but no structured home modifications	Four sessions (three visits, one booster session): Three interventions within 6–8 weeks, one booster session at 6 months, total 12 months.	Falls incidence: Self-reported calendar and phone follow-ups Functional independence: Older Americans Resources and Services ADL Scale (OARS ADL) Fear of falling: Falls Efficacy Scale International (FES-I) Health-related quality of life: 36-Item Short Form Survey (SF-36) Home safety hazards: Westmead Home Safety Assessment (WeHSA)	· Statistically significant: Falls ↓ by 39% in EG vs. CG. Self-efficacy ↑ (FES-I improved). Home modification adherence 80%. · Not statistically tested but notable: Cost-effective with 80% retention. Home modifications were well accepted and used consistently.	Effective Home modifications led to a significant reduction in falls, improved functional independence, and enhanced self-efficacy.
17	Wilson et al. (2017) [44]	Home safety assessment and modification (HSAM), including grab bars, handrails, lighting improvements, and removal of tripping hazards	Not applicable (no control group)	One-time intervention, simulation modeling conducted	Fall incidence: Self-reported falls and hospital data	· Statistically significant: HSAM reduced falls, +2800 QALYs (UI: 547–5280). Cost-effective (ICER: NZD 5480 per QALY, UI: cost-saving to NZD 15,300). Most cost-saving for 75+ individuals with prior falls. · Not statistically tested but notable: Eighty percent of homes benefited from modifications. Cost-effectiveness varied little by gender/ethnicity.	Effective Home modifications reduced falls and improved cost-effectiveness, especially for high-risk individuals with prior injurious falls.
18	Somerville et al. (2016) [45]	Occupational-therapy-based home modifications: shower seats, grab bars, lighting, and stair railings.	Not applicable (no control group)	Five sessions, one per week, over 5 weeks.	Home safety and environmental fit: In-Home Occupational Performance Evaluation (I-HOPE) Functional mobility: Performance-Oriented Mobility Assessment (Tinetti POMA) Fall risk: Informal fall history interview	· Statistically significant: I-HOPE scores ↑ post-intervention. Fall risk ↓ (Tinetti POMA improved). · Not statistically tested but notable: Common modifications: shower seats, grab bars, stair railings, and night lighting. Participants reported greater independence, less fall anxiety.	Effective Occupational-therapy-led home modifications improved functional independence, safety, and reduced fall risk.
19	Kamei et al. (2015) [32]	Home hazard modification program with safety education and hands-on training	General fall prevention program without home safety education	Four sessions, one per week, over 4 weeks, followed by follow-ups at 12 weeks (3 months) and 52 weeks (1 year)	Falls incidence: Self-reported calendar and phone follow-ups Fall prevention awareness: Custom 10-item questionnaire Home modifications compliance: 33-item checklist of home hazard assessment	· Statistically significant: Falls ↓ 10.9% overall, 11.7% indoors (52 weeks). The 75+ age group saw a significant fall reduction (12 weeks). Fall prevention awareness ↑ (*p* < 0.05). More home modifications in the HHMP group. · Not statistically tested but notable: Common modifications: clutter removal, grab bars, and non-slip mats. Fall prevention knowledge retained for 52 weeks.	Effective HHMP improved home safety behaviors and fall prevention awareness. Falls ↓ overall and indoors, especially in 75+. Home modifications were widely adopted and retained for 52 weeks.
20	Harvey et al. (2014) [46]	Home modifications (e.g., grab bars, ramps, and lighting improvements) and exercise programs (e.g., balance and strength training)	Not applicable (no control group)	One-time survey (single data collection)	Home modifications: Self-reported changes (survey) Exercise participation: Self-reported engagement (survey)	· Statistically significant: In 28.9% of cases, home modifications were made. In 35.1% of cases, exercise was increased for fall prevention. Home modifications: Age 85+ (RR 2.04), physiotherapy/OT (RR 1.57). Exercise uptake: Physiotherapy/OT (RR 2.12), Medical advice (RR 1.45). · Not statistically tested but notable: Twenty-one percent installed handrails, and five percent removed rugs. Modifications were more common in those with mobility impairments or fall history.	Correlation identified Home modifications and exercise programs were linked to fall prevention, but uptake varied by age, health status, and professional advice. More targeted strategies are needed.

↑ indicates increase; ↓ indicates decrease.

**Table 6:** Home modification details of studies.

No.	Author (Year)	Home Modification Details
1	Riera Arias et al. (2024) [33]	Mobility and accessibility improvements: Threshold removal, doorway widening, and furniture rearrangement. Bathroom safety enhancements: Non-slip mats and grab bars in shower and toilet areas. Assistive devices for daily living: Adaptive utensils, assistive tools, bed height adjustment, and bed handles. Family and caregiver education: Training on assistive devices and mobility support strategies. Fall prevention and mobility training: Space optimization, stairway, and living area safety measures.
2	Kim et al. (2024) [34]	Mobility and accessibility improvements: Addition or replacement of driveways and walkways, and door and window replacements. Flooring and interior upgrades: Installation or replacement of carpets, flooring, paneling, and ceiling tiles. Bathroom renovation: Remodeling and grab bar installation. Kitchen modification: Kitchen remodeling and optimization of cooking space. Outdoor environment improvements: Fence and wall replacements and garden and exterior repairs. Structural enhancements: Repair of main interior structures and space optimization.
3	Hawkins et al. (2024) [35]	Mobility and accessibility improvements: Grab bar installation, stair railings, and threshold removal. Bathroom safety enhancements: Grab bars in shower and toilet areas and non-slip mats. Fall prevention devices: Stair lifts and bathtub cutouts. Living space adjustments: Furniture rearrangement and pathway clearance. Walking aids: Indoor and outdoor safety railings.
4	Andersson et al. (2023) [36]	Mobility and accessibility improvements: Threshold removal, doorway widening, and installation of ramps. Bathroom safety enhancements: Grab bars in shower and toilet areas and non-slip mats. Kitchen accessibility: Relocation of overhead cabinets and shelves and workspace optimization. Outdoor accessibility: Improved access to trash bins and mailboxes and enhanced pedestrian pathways. Indoor space optimization: Furniture rearrangement and clearance around appliances.
5	Schiller et al. (2023) [37]	Mobility and accessibility improvements: Furniture rearrangement and improved doorway accessibility. Bathroom safety enhancements: Grab bar installation in showers and non-slip flooring. Home safety enhancements: Improved indoor lighting and additional stair and hallway railings. Assistive devices for daily living: Installation of electric beds and recliners and personalized mobility aids. Hazard reduction: Removal of slippery carpets and de-cluttering of unnecessary furniture.
6	Yeni et al. (2022) [38]	Mobility and accessibility improvements: Furniture rearrangement, pathway clearance, and installation of stair and hallway railings. Bathroom safety enhancements: Grab bars in bathrooms and non-slip tape application. Lighting improvements: Replacement of existing bulbs with 75W or brighter bulbs. Fall prevention measures: Securing living room and kitchen furniture and reorganizing bedroom furniture. Additional safety measures: Non-slip flooring and non-slip tape on bathtub surfaces.
7	Hollinghurst et al. (2022) [39]	Mobility and accessibility improvements: Grab bar installation, flooring replacement, and additional stair railings. Bathroom safety enhancements: Non-slip flooring and improved shower accessibility. Stair and elevation adjustments: Installation of stair lifts and ramps. Bedroom and living space enhancements: Furniture rearrangement and bed height adjustment. Home temperature regulation: Repair and improvement of boilers and central heating systems.
8	Schorderet et al. (2022) [40]	Mobility and accessibility improvements: Threshold removal, ramp installation, and improved doorway accessibility. Bathroom safety enhancements: Bathtub modification (installation of bathtub doors or conversion to a shower) and grab bar installation. Home safety enhancements: Lighting improvements (brightness adjustment and sensor lighting) and electrical safety enhancements. Kitchen and interior improvements: Kitchen remodeling (adjusting appliance height and modifying storage spaces) and improved balcony accessibility. Convenience enhancements: Installation of automatic blinds and addition of a video intercom system.
9	Malmgren Fänge et al. (2021) [41]	Mobility and accessibility improvements: Threshold removal, doorway widening, and secured movement pathways. Bathroom safety enhancements: Grab bar installation around toilets and showers and non-slip flooring application. Indoor mobility enhancements: Additional stair railings and furniture rearrangement for easier movement. Kitchen accessibility improvements: Adjusted cabinet heights and optimized cooking space. Fall prevention: Replacement of slippery flooring and improved lighting brightness.
10	Stasi et al. (2021) [29]	Mobility and accessibility improvements: Threshold removal, doorway widening, and furniture rearrangement for clear pathways. Bathroom safety enhancements: Installation of non-slip mats and grab bars around showers and toilets. Lighting and visual accessibility: Replacement of existing bulbs with high-intensity LED lights for better visibility. Fall prevention and mobility training: Installation of handrails in stairways and living areas and space optimization. Kitchen and living space improvements: Adjustment of high cabinets and enhanced accessibility to sinks and cooking areas. Outdoor environment enhancements: Repair of driveways and walkways and addition of railings and ramps. Bedroom adjustments: Bed height modification, installation of bed handles, and addition of night lighting.
11	Tsekoura et al. (2021) [30]	Mobility and accessibility improvements: Threshold removal, furniture rearrangement, and enhanced doorway accessibility. Bathroom safety enhancements: Installation of grab bars around showers and toilets and application of non-slip flooring. Lighting and visual accessibility: Replacement of existing bulbs with bright LED lights and installation of motion sensor lighting. Fall prevention and mobility training: Addition of stair and hallway handrails and application of non-slip flooring. Kitchen accessibility improvements: Optimization of cooking space and adjustment of cabinet heights. Outdoor environment enhancements: Driveway and walkway repairs and installation of ramps.
12	Jeon et al. (2020) [31]	Mobility and accessibility improvements: Threshold removal, doorway widening, and furniture rearrangement for easier movement. Bathroom safety enhancements: Installation of grab bars around showers and toilets and application of non-slip flooring. Home safety enhancements: Improved indoor lighting (brightness adjustment and motion sensor lights) and addition of stair and hallway handrails. Utilization of assistive devices: Provision of customized assistive devices (e.g., mobility aids and adaptive utensils), bed height adjustment, and installation of bed handles. Education for family and caregivers: Training on the use of assistive devices and mobility support strategies and guidance on creating an independence-supportive environment. Fall prevention and mobility training: Optimization of space to reduce fall risks and reinforcement of safety measures in stairways and living areas.
13	Carnemolla et al. (2019) [42]	Mobility and accessibility improvements: Doorway widening and installation of ramps for better entrance and stair accessibility. Bathroom safety enhancements: Grab bars around showers and toilets, application of non-slip flooring. Home safety enhancements: Improved indoor lighting (motion sensors and adjustable brightness) and addition of stair and hallway handrails. Kitchen and laundry adjustments: Height adjustment of countertops and washing machines and reorganization of storage for better accessibility. Fall prevention measures: Application of non-slip flooring, furniture rearrangement, and removal of obstacles. Support for independent living: Installation of personalized mobility assistive devices.
14	Wilson et al. (2019) [43]	Mobility and accessibility improvements: Doorway widening, threshold removal, and furniture rearrangement for improved movement. Bathroom safety enhancements: Grab bars around showers and toilets and application of non-slip flooring. Home safety enhancements: Installation of sensor lighting and adjustable-brightness lights and addition of stair and hallway handrails. Kitchen and living space adjustments: Height adjustment of countertops and washing machines and reorganization of storage for better accessibility. Fall prevention measures: Application of non-slip flooring, removal of obstacles, and optimized furniture placement. Support for independent living: Installation of personalized mobility assistive devices (e.g., wheelchair-accessible furniture arrangements).
15	Pettersson et al. (2018) [14]	Mobility and accessibility improvements: Threshold removal, doorway widening, and installation of ramps for entrance and stair access. Bathroom safety enhancements: Grab bars around showers and toilets and conversion of bathtubs into walk-in showers. Home safety enhancements: Sensor lighting installation, adjustable-brightness lighting, and addition of stair and hallway handrails. Kitchen and living space adjustments: Height adjustment of countertops and washing machines and reorganization of storage for better accessibility. Fall prevention measures: Application of non-slip flooring, removal of obstacles, and optimized furniture placement. Support for independent living: Furniture arrangement accommodating wheelchair and mobility aid users.
16	Stark et al. (2017) [21]	Mobility and accessibility improvements: Threshold removal, doorway widening, and furniture rearrangement for improved movement. Bathroom safety enhancements: Installation of grab bars around showers and toilets and application of non-slip flooring. Home safety enhancements: Improved indoor lighting (adjustable brightness and sensor lights) and addition of stair and hallway handrails. Kitchen and living space adjustments: Height adjustment of countertops and washing machines and optimization of storage and appliance placement. Fall prevention measures: Application of non-slip flooring, removal of obstacles, and optimized furniture arrangement. Support for independent living: Provision of personalized mobility assistive devices.
17	Wilson et al. (2017) [44]	Mobility and accessibility improvements: Threshold removal, doorway widening, and furniture rearrangement to enhance movement. Bathroom safety enhancements: Installation of grab bars around showers and toilets and application of non-slip flooring. Lighting improvements: Addition of sensor lights in dark hallways and stairs and installation of adjustable-brightness lighting. Stair safety enhancements: Installation of handrails, application of anti-slip tape, and improved stair lighting. Kitchen and living space modifications: Adjustment of countertop and storage heights and optimized layout for accessibility. Fall prevention measures: Removal of indoor hazards and application of non-slip flooring in living rooms and bedrooms. Support for independent living: Furniture arrangement for wheelchair accessibility and installation of assistive devices.
18	Somerville et al. (2016) [45]	Mobility and accessibility improvements: Furniture rearrangement to secure movement pathways and installation of handrails in stairs and hallways. Bathroom safety enhancements: Installation of grab bars around showers and toilets and addition of a foldable shower chair. Lighting improvements: Sensor-activated night lighting in bedrooms and hallways. Stair safety enhancements: Addition of handrails in basement stairs and application of non-slip flooring. Fall prevention measures: Removal of indoor hazards and application of non-slip flooring in living rooms and bedrooms. Support for independent living: Improved doorway accessibility and installation of personalized mobility assistive devices.
19	Kamei et al. (2015) [32]	Mobility and accessibility improvements: Removal of thresholds, widening of doorways, and furniture rearrangement to secure movement pathways. Bathroom safety enhancements: Application of non-slip flooring and installation of grab bars around showers and toilets. Lighting improvements: Addition of night lighting in stairways and hallways and installation of adjustable-brightness lighting. Stair safety enhancements: Installation of handrails, application of non-slip tape, and improved stair lighting. Kitchen and living space modifications: Adjustment of counter and storage heights for better accessibility and optimized layout. Fall prevention measures: Removal of household hazards and application of non-slip flooring in living rooms and bedrooms. Support for independent living: Furniture arrangement for wheelchair accessibility and installation of assistive devices.
20	Harvey et al. (2014) [46]	Mobility and accessibility improvements: Removal of thresholds, widening of doorways, and furniture rearrangement to secure movement pathways. Bathroom safety enhancements: Installation of grab bars around showers and toilets and application of non-slip flooring. Lighting improvements: Addition of night lighting in stairways and hallways and installation of adjustable-brightness lighting. Stair safety enhancements: Installation of handrails, application of non-slip tape, and improved stair lighting. Kitchen and living space modifications: Adjustment of counter and storage heights for better accessibility and optimized layout. Fall prevention measures: Removal of household hazards and application of non-slip flooring in living rooms and bedrooms. Support for independent living: Furniture arrangement for wheelchair accessibility and installation of assistive devices.

**Table 7:** A multidimensional analysis of home modification for aging in place among older adults.

Analysis Items	Study
Effectiveness of Home Modification for Fall Prevention (Total: 12 studies)	Stasi et al. (2021) [29], Tsekoura et al. (2021) [30], Stark et al. (2017) [21], Kamei et al. (2015) [32], Hawkins et al. (2024) [35], Andersson et al. (2023) [36], Yeni et al. (2022) [38], Hollinghurst et al. (2022) [39], Malmgren Fänge et al. (2021) [41], Wilson et al. (2017) [44], Somerville et al. (2016) [45], and Harvey et al. (2014) [46]
Effect of Home Modifications on Enhancing Independence in Activities of Daily Living (Total: 6 studies)	Jeon et al. (2020) [31], Stark et al. (2017) [21], Riera Arias et al. (2024) [33], Yeni et al. (2022) [38], Schorderet et al. (2022) [40], and Malmgren Fänge et al. (2021) [41]
Impact of Home Modification on Quality of Life in Older Adults (Total: 5 studies)	Stasi et al. (2021) [29], Tsekoura et al. (2021) [30], Jeon et al. (2020) [31], Riera Arias et al. (2024) [33], and Schorderet et al. (2022) [40]
Impact of Home Modifications on Home Safety (Total: 4 studies)	Stark et al. (2017) [21], Schiller et al. (2023) [37], Wilson et al. (2019) [43], and Somerville et al. (2016) [45]
Impact of Home Modifications on Housing Accessibility (Total: 2 studies)	Andersson et al. (2023) [36] and Pettersson et al. (2018) [14]
Home Modification for Older Adults with Cognitive Decline (Total: 2 studies)	Jeon et al. (2020) [31] and Yeni et al. (2022) [38]
Impact of Home Modification on Reducing Caregiving Burden (Total: 2 studies)	Jeon et al. (2020) [31] and Carnemolla et al. (2019) [42]
Cost-Effectiveness Analysis of Home Modification (Total: 2 studies)	Stark et al. (2017) [21] and Wilson et al. (2017) [44]
Disparities in Home Modification Accessibility by Socioeconomic Factors (Total: 2 studies)	Kim et al. (2024) [34] and Hollinghurst et al. (2022) [39]

**Table 8:** Integrated analysis of health changes and home modification among older adults.

Analysis Items	Study	Key Findings	Analysis Summary
Physical Changes and Home Modification	Jeon et al. (2020) [31]	Improved functional independence (*p* < 0.05) and quality of life (*p* < 0.05). Slight increase in caregiver burden despite improvements in function and quality of life.	·Physical changes due to aging (muscle weakness, balance changes, and functional decline) interact with home modification, requiring continuous and systematic interventions. - Structural modifications addressing muscle weakness and balance changes improve mobility. - Mobility maintenance is a key factor for aging in place. - Home modifications for fall prevention (e.g., grab bars, non-slip flooring, and path clearing) significantly reduce fall rates and improve quality of life. - - Fall reduction leads to medical cost savings, emphasizing the economic need for home modification.
	Andersson et al. (2023) [36]	Parkinson’s disease housing accessibility changed over time; grab bars and stair replacements helped, but new outdoor barriers emerged, requiring ongoing modifications.	
	Hollinghurst et al. (2022) [39]	A 3% reduction in fall-related emergency hospitalizations (OR = 0.97, *p* < 0.001); frailty and socioeconomic deprivation increased fall risk. Home modifications reduced fall-related emergency hospitalizations, especially in older adults with prior falls.	
	Pettersson et al. (2018) [14]	Increased accessibility in older and single-family homes. Removing environmental barriers reduced accessibility issues by up to 35%. Systematic home modifications significantly improved housing accessibility for older adults.	
	Wilson et al. (2017) [44]	Reduced falls and medical costs and increased quality of life (QALY). Greatest cost-saving effects were observed in individuals aged 75+ and those with prior fall experiences. Home modifications reduced falls and were highly cost-effective, particularly in high-risk groups with previous fall experiences.	
Maintenance of Physical Function and Balance with Home Modification	Stasi et al. (2021) [29]	Increased mobility (TUG test, *p* < 0.001), improved balance (Tandem stance, *p* < 0.001), improved quality of life (EQ-5D-5L VAS, *p* < 0.001), and reduced fear of falling (FES-I, *p* = 0.001) after combined home modification and exercise intervention.	· Home modification combined with exercise is essential for maintaining physical function and improving balance. - Physical function maintenance and balance improvement maximize mobility and fall prevention. - Physical modifications, such as grab bars and stair railings, play a critical role. - Maintaining physical function enhances independent living and aging in place. - Combining home modification and exercise effectively reduces falls and improves quality of life. - - An integrated approach is necessary for effective fall prevention and independent living.
	Tsekoura et al. (2021) [30]	Reduced falls after the intervention. Improved mobility (TUG, *p* < 0.001) and balance (*p* < 0.001). Home modification and exercise interventions were effective, with grab bars and stair railings playing a significant role.	
Cognitive Function Changes and Home Modification	Jeon et al. (2020) [31]	Increased functional independence (*p* < 0.05) and improved quality of life (*p* < 0.05). Improvement in function and quality of life, but a slight increase in caregiver burden. Gradual adjustments to maintain familiar environments and enhance safety are more effective than abrupt changes. Grab bars, railings, and furniture rearrangement improved mobility and prevented falls. Effectiveness increased when combined with cognitive rehabilitation programs.	· Home modification for individuals with cognitive decline should focus on maintaining a familiar environment while enhancing safety to prevent falls and behavioral issues and improve functional independence. - Fall prevention and functional independence improvement: Modifications in high-risk areas like bathrooms and toilets are particularly effective. - Maintaining familiar environments is crucial: Enhancing safety in the existing environment is preferable to introducing new changes. - Support for memory retention and spatial awareness: Repetitive usage patterns help maintain memory and spatial recognition. - Reducing behavioral issues and fall risk: Adjustments should enhance safety without abruptly changing the environment. - - Reducing caregiver burden: While effective, home modification may increase caregiver burden, requiring additional support measures.
	Yeni et al. (2022) [38]	No falls in modified environments (*p* = 0.000) and fall reduction after 3 months (*p* = 0.002). Home modifications for individuals with dementia were effective in preventing falls, especially in bathrooms and toilets. Main fall causes: slipping and loss of balance; grab bars and non-slip mats were effective in fall prevention. Interventions should enhance safety while maintaining familiar environments. Repeated use in familiar spaces helps with memory and spatial awareness, reducing home hazards and facilitating caregiver collaboration when necessary.	

## Data Availability

The article includes all data supporting the reported results; further details on the methodology are available from the corresponding author upon reasonable request.

## References

[1] United Nations, Department of Economic and Social Affairs, Population Division (2019). World Population Ageing 2019: Highlights.

[2] United Nations, Department of Economic and Social Affairs, Population Division (2023). World Social Report 2023: Leaving No One Behind in an Ageing World.

[3] Sixsmith J., Sixsmith A. (2008). Aging in place in the United Kingdom. Aging Int..

[4] Harrell R., Lynott J., Guzman S., Lampkin C. (2014). What Is Livable? Community Preferences of Older Adults.

[5] Vespa J., Engelberg J., He W. (2020). Old Housing, New Needs: Are U.S. Homes Ready for an Aging Population?.

[6] Leidelmeijer K., Iersel J.V., Leering D. (2017). Monitor Investeren in de Toekomst–Ouderen en Langer Zelfstandig Wonen.

[7] de Klerk M., Verbeek-Oudijk D., Plaisier I., den Draak M. (2019). Zorgen voor Thuiswonende Ouderen.

[8] Braubach M., Power A. (2011). Housing conditions and risk: Reporting on a European study of housing quality and risk of accidents for older people. J. Hous. Elder..

[9] Ratnayake M., Lukas S., Brathwaite S., Neave J., Henry H. (2022). Aging in place: Are we prepared?. Del. J. Public Health.

[10] Gitlin L.N. (2003). Conducting research on home environments: Lessons learned and new directions. Gerontologist.

[11] Lee S.H., Yu S. (2020). Effectiveness of multifactorial interventions in preventing falls among older adults in the community: A systematic review and meta-analysis. Int. J. Nurs. Stud..

[12] Gillespie L.D., Robertson M.C., Gillespie W.J., Sherrington C., Gates S., Clemson L.M., Lamb S.E. (2012). Interventions for preventing falls in older people living in the community. Cochrane Database Syst. Rev..

[13] Wahl H.W., Fänge A., Oswald F., Gitlin L.N., Iwarsson S. (2009). The home environment and disability-related outcomes in aging individuals: What is the empirical evidence?. Gerontologist.

[14] Pettersson C., Slaug B., Granbom M., Kylberg M., Iwarsson S. (2018). Housing accessibility for senior citizens in Sweden: Estimation of the effects of targeted elimination of environmental barriers. Scand. J. Occup. Ther..

[15] Nicklett E., Lohman M., Smith M. (2017). Neighborhood environment and falls among community-dwelling older adults. Int. J. Environ. Res. Public Health.

[16] Runyan C.W., Casteel C., Perkis D., Black C., Marshall S.W., Johnson R.M., Waller A.E., Viswanathan S. (2005). Unintentional injuries in the home in the United States Part I: Mortality. Am. J. Prev. Med..

[17] Gaugler J.E., Duval S., Anderson K.A., Kane R.L. (2007). Predicting nursing home admission in the U.S: A meta-analysis. BMC Geriatr..

[18] Freedman V.A., Spillman B.C. (2014). The residential continuum from home to nursing home: Size, characteristics and unmet needs of older adults. J. Gerontol. B Psychol. Sci. Soc. Sci..

[19] Sophonratanapokin B., Sawangdee Y., Soonthorndhada K. (2012). Effect of the living environment on falls among the elderly in Thailand. Southeast Asian J. Trop. Med. Public Health.

[20] Zhang L., Ding Z., Qiu L., Li A. (2019). Falls and risk factors of falls for urban and rural community-dwelling older adults in China. BMC Geriatr..

[21] Stark S., Somerville E., Keglovits M., Conte J., Li M., Hu Y.L., Yan Y. (2017). Protocol for the home hazards removal program (HARP) study: A pragmatic, randomized clinical trial and implementation study. BMC Geriatr..

[22] Gitlin L.N. (1998). Testing home modification interventions: Issues of theory, measurement, design, and implementation. Annu. Rev. Gerontol. Geriatr..

[23] Siebert C., Smallfield S., Stark S. (2014). Occupational Therapy Practice Guidelines for Home Modifications.

[24] Byrnes M., Lichtenberg P.A., Lysack C. (2006). Environmental Press, Aging in Place, and Residential Satisfaction of Urban Older Adults. J. Appl. Sociol./Sociol. Pract..

[25] Natalia M., Yang S.L., Chen Y.J., Tzu H.H., Liao C.P., Huang C.H., Chou Y.C. (2023). Person–Environment–Occupation Model in the Quality Improvement and Patient Safety Education: A Case Study. Clin. Case Rep. Int..

[26] Moseley A.M., Herbert R.D., Sherrington C., Maher C.G. (2002). Evidence for physiotherapy practice: A survey of the Physiotherapy Evidence Database (PEDro). Aust. J. Physiother..

[27] Slim K., Nini E., Forestier D. (2003). Methodological index for non-randomized studies (minors): Development and validation of a new instrument. ANZ J. Surg..

[28] Braga L.H., Mijovic H., Farrokhyar F., Pemberton J., DeMaria J., Lorenzo A.J. (2013). Antibiotic prophylaxis for urinary tract infections in antenatal hydronephrosis. Pediatrics.

[29] Stasi S., Tsekoura M., Gliatis J., Sakellari V. (2021). Motor control and ergonomic intervention home-based program: A pilot trial performed in the framework of the motor control home ergonomics elderlies’ prevention of falls. Cureus.

[30] Tsekoura M., Stasi S., Gliatis J., Sakellari V. (2021). Methodology of a home-based motor control exercise and ergonomic intervention programme for community-dwelling older people: The McHeELP study. J. Frailty Sarcopenia Falls.

[31] Jeon Y.H., Krein L., Simpson J.M., Szanton S.L., Clemson L., Naismith S.L., Low L.-F., Mowszowski L., Gonski P., Norman R. (2020). Feasibility and potential effects of interdisciplinary home-based reablement program (I-HARP) for people with cognitive and functional decline: A pilot trial. Aging Ment. Health.

[32] Kamei T., Kajii F., Yamamoto Y., Irie Y., Kozakai R., Sugimoto T., Chigira A., Niino N. (2015). Effectiveness of a home hazard modification program for reducing falls in urban community-dwelling older adults: A randomized controlled trial. JPN. J. Nurs. Sci..

[33] Riera Arias G., Serra Corcoll J., Casadevall Arnaus M., Vidal-Alaball J., Ramírez-Morros A., Arnau Solé G. (2024). Improving quality of life in older adults with the decline syndrome: The role of occupational therapy in primary care. Aten. Primaria.

[34] Kim D., Lee M.J., Kang J. (2024). Exploring differences in home modification strategies according to household location and occupant disability status: 2019 American Housing Survey analysis. J. Appl. Gerontol..

[35] Hawkins M., Goldhammer T., McClave R., Jenkins-Smith E. (2024). Evaluation of a fall prevention program to reduce fall risk and fear of falling among community-dwelling older adults and adults with disabilities. Clin. Interv. Aging.

[36] Andersson N., Slaug B., Nilsson M.H., Iwarsson S. (2023). Environmental barriers and housing accessibility problems for people with Parkinson’s disease: A three-year perspective. Scand. J. Occup. Ther..

[37] Schiller G., Seligman A., Lubetsky S., DeCherrie L.V., Reckrey J., Kopke V., Bacher N., Bhatia S., Ornstein K.A. (2023). A home repair and modification program embedded within Mount Sinai Visiting Doctors. J. Appl. Gerontol..

[38] Yeni C., Yilmaz M. (2022). Nurse-led home modification interventions for community-dwelling older adults with dementia and their impact on falls prevention. Br. J. Healthc. Assist..

[39] Hollinghurst J., Daniels H., Fry R., Akbari A., Rodgers S., Watkins A., Hillcoat-Nallétamby S., Williams N., Nikolova S., Meads D. (2022). Do home adaptation interventions help to reduce emergency fall admissions? A national longitudinal data-linkage study of 657,536 older adults living in Wales (UK) between 2010 and 2017. Age Ageing.

[40] Schorderet C., Ludwig C., Wüest F., Bastiaenen C.H.G., de Bie R.A., Allet L. (2022). Needs, benefits, and issues related to home adaptation: A user-centered case series applying a mixed-methods design. BMC Geriatr..

[41] Malmgren Fänge A., Chiatti C., Axmon A. (2021). One-year changes in activities of daily living, usability, falls and concerns about falling, and self-rated health for different housing adaptation client profiles. Int. J. Environ. Res. Public Health.

[42] Carnemolla P., Bridge C. (2019). Housing design and community care: How home modifications reduce care needs of older people and people with disability. Int. J. Environ. Res. Public Health.

[43] Wilson G., Aitken D., Hodgson P., Bailey C. (2019). The hidden impact of home adaptations: Using a wearable camera to explore lived experiences and taken-for-granted behaviours. Health Soc. Care Community.

[44] Wilson N., Kvizhinadze G., Pega F., Nair N., Blakely T. (2017). Home modification to reduce falls at a health district level: Modeling health gain, health inequalities and health costs. PLoS ONE.

[45] Somerville E., Smallfield S., Stark S., Seibert C., Arbesman M., Lieberman D. (2016). Occupational therapy home modification assessment and intervention. Am. J. Occup. Ther..

[46] Harvey L.A., Mitchell R.J., Lord S.R., Close J.C.T. (2014). Determinants of uptake of home modifications and exercise to prevent falls in community-dwelling older people. Aust. N. Z. J. Public Health.

[47] De Morton N.A. (2009). The PEDro scale is a valid measure of the methodological quality of clinical trials: A demographic study. Aust. J. Physiother..

[48] Paez A. (2017). Gray literature: An important resource in systematic reviews. J. Evid.-Based Med..

[49] Iwarsson S., Slaug B. (2001). Housing Enabler: An Instrument for Assessing and Analyzing Accessibility Problems in Housing.

[50] Clemson L., Fitzgerald M.H., Heard R. (1999). Content validity of an assessment tool to identify home fall hazards: The Westmead Home Safety Assessment. Br. J. Occup. Ther..

[51] Higgins J.P., Thomas J., Chandler J., Cumpston M., Li T., Page M.J., Welch V.A. (2022). Cochrane Handbook for Systematic Reviews of Interventions.

[52] Higgins J.P.T., Thompson S.G., Deeks J.J., Altman D.G. (2003). Measuring inconsistency in meta-analyses. BMJ.

[53] Altman D.G. (2001). Practical Statistics for Medical Research.

[54] Sterne J.A.C., Hernán M.A., Reeves B.C., Savović J., Berkman N.D., Viswanathan M., Higgins J.P.T. (2016). ROBINS-I: A Tool for Assessing Risk of Bias in Non-Randomised Studies of Interventions. BMJ.

[55] Lee C., Coughlin J.F. (2015). Older Adults’ Adoption of Technology: An Integrated Approach to Identifying Determinants and Barriers. J. Prod. Innov. Manag..

[56] Stark S., Keglovits M., Arbesman M., Lieberman D. (2017). Effect of Home Modification Interventions on the Participation of Community-Dwelling Adults with Health Conditions: A Systematic Review. Am. J. Occup. Ther..

[57] Peek S.T., Wouters E.J., van Hoof J., Luijkx K.G., Boeije H.R., Vrijhoef H.J. (2014). Factors influencing acceptance of technology for aging in place: A systematic review. Int. J. Med. Inform..

[58] Chase C.A., Mann K., Wasek S., Arbesman M. (2012). Systematic Review of the Effect of Home Modification and Fall Prevention Programs on Falls and the Performance of Community-Dwelling Older Adults. Am. J. Occup. Ther..

[59] Arora A., Mahajan H. (2024). Impact of Built Environment on Fear of Falls among Older Adults: A Systematic Review. Arch. Phys. Med. Rehabil..

[60] Pillay J., Gaudet L.A., Saba S., Vandermeer B. (2024). Falls prevention interventions for community-dwelling older adults: Systematic review and meta-analysis of benefits, harms, and patient values and preferences. Syst. Rev..

[61] Sourial N., Beuscart J.B., Posłuszny Ł., Calafiore M., Sousa S.S., Sansone E., Zuber M., Vedel I., COVERAGE Collaborative Group (2024). Challenges and solutions in recruiting older vulnerable adults in research. Int. J. Public Health..

[62] Damme M.J., Ray-Degges S. (2016). A Qualitative Study on Home Modification of Rural Caregivers for People with Dementia. J. Hous. Elder..

[63] Yang S.Y., Fu S.H., Hsieh P.L., Lin Y.L., Chen M.C., Lin P.H. (2022). Improving the care stress, life quality, and family functions for family-caregiver in long-term care by home modification. Ind. Health.

[64] Aliberti M.J.R., Covinsky K.E. (2019). Home Modifications to Reduce Disability in Older Adults with Functional Disability. JAMA Intern. Med..

[65] Keglovits M., Stark S. (2020). Home Modifications to Improve Function and Safety in the United States. J. Aging Environ..

[66] Nagasawa S. (2015). Long-term Care Insurance Act and Home Care. JMAJ.

[67] Rubenstein L.Z., Josephson K.R. (2006). Falls and Their Prevention in Elderly People: What Does the Evidence Show?. Med. Clin. North Am..

[68] Wang J., Li Y., Yang G.-Y., Jin K. (2024). Age-Related Dysfunction in Balance: A Comprehensive Review of Causes, Consequences, and Interventions. Aging Dis..

[69] Ganz D.A., Latham N.K. (2020). Prevention of Falls in Community-Dwelling Older Adults. N. Engl. J. Med..

[70] Sturnieks D.L., St George R., Lord S.R. (2008). Balance Disorders in the Elderly. Neurophysiol. Clin..

[71] Pizzigalli L., Filippini A., Ahmaidi S., Jullien H., Rainoldi A. (2011). Prevention of Falling Risk in Elderly People: The Relevance of Muscular Strength and Symmetry of Lower Limbs in Postural Stability. J. Strength Cond. Res..

[72] Pynoos J., Steinman B.A., Nguyen A.Q.D. (2010). Environmental Assessment and Modification as Fall-Prevention Strategies for Older Adults. Clin. Geriatr. Med..

[73] Montero-Odasso M. (2018). Falls in Cognitively Impaired Older Adults: Implications for Risk Assessment and Prevention. J. Am. Geriatr. Soc..

